# Comparing Anti-hepatitis B Antibody Level in Iranian Obese or Overweight with Non-obese Cases

**DOI:** 10.18869/acadpub.ibj.21.3.197

**Published:** 2017-05

**Authors:** Ali Kabir, Abdolreza Pazouki, Motahareh Jafari, Somayye Mokhber, Mohammad Vaziri, Seyed Moayed Alavian

**Affiliations:** 1 Minimally Invasive Surgery Research Center, Iran University of Medical Sciences, Tehran, Iran; 2 Middle East Liver Disease Center, Tehran, Iran

**Keywords:** Hepatitis B vaccines, Obesity, Liver diseases, Immunosuppressive agent

## Abstract

**Background:**

There is a controversy about the relation between anti-hepatitis B (anti-HBs) antibody level and obesity. We designed this study to compare the vaccine efficacy in obese/overweight and non-obese cases.

**Methods:**

In this cross-sectional study, 242 obese/overweight and 85 non-obese individuals were participated. Cases were selected from a referral clinic for obesity and a referral hepatology clinic, both in Tehran, Iran.

**Results:**

Obese cases had lower percentage of liver diseases (66.9% vs. 100%, *P*<0.001) but higher percentage of hepatitis B vaccination history (74.9% vs. 51.2%, *P*<0.001). Median±inter-quartile range of anti-HBs titer in obese cases was significantly lower than controls (48.5±194.5 vs. 100±557.6, *P*=0.012).

**Conclusion:**

The level of anti-HBs surface antigen antibody’s titer in obese cases without liver disease is lower than control group. Therefore, a suitable strategy is needed to overcome this problem, which can be the use of longer needles for vaccination.

## INTRODUCTION

Iran is among countries with an intermediate prevalence of hepatitis B virus (HBV)[1]. Vaccination is a very suitable strategy for preventing HBV infection[2]. A protective level of anti-hepatitis B (anti-HBs) is necessary to assure the preventive effect of vaccination[2]. However, cases with poor response to hepatitis B vaccine should be considered by using new vaccination strategies. To develop such new strategies, we should first determine the possible causes of insufficient response to vaccination. One of the probable non-responder groups to hepatitis B vaccine is the group of obese cases.

Based on the two systematic reviews, the prevalence of obesity is between 12.6% and 25.9%[3,4] in Iranian adult population, while in many countries, it is more than 50%.

Two previous studies on public safety personnel[5] or hospital staffs[6] have shown that higher body mass index (BMI) is a marker of poor response to hepatitis B vaccine. Two others have indicated that the gluteal injection of hepatitis B vaccine results in lower immunogenictity compare to deltoid injection, which is supposed to be related to the higher amount of fat tissue in gluteal region[7,8].

In most previous studies that have evaluated the role of BMI as a risk factor for response to hepatitis B vaccine, their main goal was not assessing BMI as the main exposure. However, they aimed at evaluating the role of BMI beside many other risk factors (like cross-sectional studies) and not as a main factor (like case-control or cohort studies). Furthermore, they just examined multiple factors such as age, sex, BMI, and comorbidities[5,6]. Here, we have designed a control group with normal BMI to compare with obese and overweight cases. Considering the prevalence of HBV and the increasing trend of obesity in Iran, the present study was designed to compare the vaccine efficacy in obese/overweight and non-obese cases.

## MATERIALS AND METHODS

In this cross-sectional study, 242 obese and 85 non-obese individuals were participated between March 2013 and March 2014. Some cases were selected from a referral clinic for obesity, and some other cases as well as all controls were chosen from a referral hepatology clinic, both in Tehran, Iran.

Cases were individuals with BMI equal to or higher than 25 and controls with BMI lower than 25 kg/m^2^, respectively. Controls were selected from referral cases with non-alcoholic fatty liver disease, auto-immune hepatitis, primary biliary cirrhosis, and primary sclerosing cholangitis. The controls were free of viral hepatitis B or C and HIV. In other words, any liver diseases that did not have any effect on the presence or the level of HBsAb were considered as control cases.

Demographic data of both cases and controls were obtained from the profile of both groups that included age, gender, education, vaccination history, comorbidities, immunosuppressive medication, liver disease in participants or their families, height, weight, BMI, and HBV profile (consist of anti-HBs titer)Anti-HBs titer equal to or higher than 10 mIU/mL was considered positive.

We estimated the sample size of 242 in cases and 88 in controls based on two-sample comparison of means formula, considering type I and II errors, mean and standard deviation of anti-HBs titer in the case and control groups and the ratios of the case group to the control were equal to 0.05, 0.1, 160, 110, 120, 60, and 2.75, respectively. However, we did not find more than 85 controls.

### Ethics

The study was approved by the ethics committee of Iran University of Medical Sciences, Iran, Tehran. The study protocol was also conformed to the ethical guidelines of the 1975 Declaration of Helsinki as reflected in a prior approval by the institution’s human research committee. An informed consent was obtained from all participants for using their data in research projects anonymously.

### Statistical analysis

Data analysis was performed based on mean±standard deviation (SD), median±interquartile range (IQR), percentage, odds ratio (OR) with 95% confidence interval (95% CI), chi-square, Mann-Whitney U, Kruskal Wallis H, Median test, Spearman’s rho correlation coefficient, logistic (Forward-Wald model), and linear regression (stepwise model) using SPSS 20.0 (SPSS Inc., Chicago, Illinois, USA). *P* value less than 0.05 was considered statistically significant. In regression analysis, all variables with *P*<0.2 were entered in the model if they had confounder role in the relation between the level of anti-HBs titer and BMI category (≥25 or <25 kg/m^2^).

## RESULTS

The mean±SD age of the participants was 42±12.9 years, and 202 out of 327 cases (61.8%) were female. We observed that 83 obese cases (43.7%) responded to vaccination, without any significant difference, versus 15 (34.9%) non-obese ones. None of the factors, including sex, age, height, immunosuppressive medication usage, family history of liver diseases, type of liver diseases, SGOT, SGPT, and alkaline phosphatase were associated with response to vaccination. Cases with the presence of liver disease had significantly higher response to vaccination (OR: 3, 95% CI: 1.4, 6.6; *P*=0.004). Mean weight (93.4±26 vs. 113.2±31.1 kg, *P*<0.001) and BMI (33.8±8.8 vs. 41.1±10 kg/m^2^, *P*<0.001) were lower in cases with response to vaccination. The comparison between BMI and the presence of liver disease indicated that only BMI (adjusted OR: 0.92, 95% CI: 0.88, 0.96; *P*<0.001) had significant association with response to vaccination. Higher BMI caused a lower response rate as OR was significantly lower than 1.

Mean (*P*=0.011) but not the median of the level of anti-HBs titer decreased significantly by an increase in BMI. Means (SD) of BMI categories, including 18-24.9, 25-29.9, 30-34.9, and >35 kg/m^2^ were equal to 279.4 (393.7), 241.9 (337.3), 195.2 (271.6), and 70.0 (164.4) mIU/mL, respectively. The sex and family history of liver disease were not different between the obese group and the controls. The non-obese group had significantly higher immunosuppressive medication usage (*P*<0.001) and liver diseases (*P*<0.001) but lower age (*P*=0.046) (Table 1). The type of liver disease was mostly non-alcoholic fatty liver disease in cases, while autoimmune hepatitis in controls (*P*<0.001). The pattern of vaccination was also different between the two groups (*P*<0.001). Among individuals with the specified history of vaccination, 146 (74.9%) of obese group and 21 (51.2%) of non-obese one had vaccination against hepatitis B (Table 1). In brief, cases and controls were significantly different in view of immunosuppressive medication usage, liver diseases, age, type of liver disease, and pattern of vaccination. Therefore, association between these probable confounders and anti-HBs titer was evaluated in the next step. Titer of anti-HBs was significantly higher in patients with liver diseases and higher numbers of vaccination times but it was not related to sex, taking immunosuppressive medications, family history of liver disease, and type of liver disease (Table 2). Age was not correlated with the level of anti-HBs titer (*P*=0.581). Median±IQR of anti-HBs titer in obese cases was significantly lower than controls (48.5±194.5 vs. 100±557.6 mIU/mL, *P*=0.012). BMI had a reverse moderate correlation with the level of anti-HBs titer (r=-0.379, *P*<0.001).

**Table 1:** Basic Characteristics of obese and non-obese individuals

Characteristics	Cases (n=242)	Controls (n=85)
Age, year, mean±SD	42.9±12.1	39.4±14.7
Female, no. (%)	149 (61.6)	53 (62.4)
Liver disease, no. (%)	162 (66.9)	85 (100)
Type of liver disease, no. (%)		
NAFLD	118 (73.3)	22 (25.9)
Autoimmune hepatitis	33 (20.5)	47 (55.3)
PBC/PSC	10 (6.2)	16 (18.8)
Family history of liver disease, no. (%)	29 (12.2)	4 (4.7)
Vaccination history, no. (%)		
No	49 (20.2)	20 (23.5)
One time	9 (3.7)	1 (1.2)
Two times	11 (4.5)	0
Three times	126 (52.1)	20 (23.5)
Unknown	47 (19.4)	44 (51.8)
Immunosuppressive medication, no. (%)	25 (10.3)	34 (40)

NAFLD, non-alcoholic fatty liver disease; no., number; PBC, primary biliary cirrhosis; PSC, primary sclerosing cholangitis; SD, standard deviation

**Table 2:** Anti-hepatitis Bs titer level in different subgroups

Characteristic	Anti-HBs level, mIU/mL, median±IQR	*P* value
Sex		
Male	54±273.5	0.983
Female	49±174	
Immunosuppressive usage			
Yes	100±968	0.232
No	48.5±185.5	
Liver disease			
Yes	86±298.7	0.023
No	22±92	
Type of liver disease			
NAFLD	86±271.5	0.258
Autoimmune hepatitis	100±968	
PBC/PSC	45±22	
Family history of liver disease			
Yes	96±548.8	0.799
No	50±161	
Vaccination history			
No	39±328.4	0.035
One time	5.5±32.8	
Two times	2±9	
Three times	64.5±223.2		
Unknown	49.5±92		

HBs, hepatitis B surface; IQR, inter-quartile range; mIU, mili international unit; mL, milliliter; NAFLD, non-alcoholic fatty liver disease; PBC, primary biliary cirrhosis; PSC, primary sclerosing cholangitis.

In the present study, confounder variables in association between anti-HBs titer and BMI group (obese vs. non-obese) are liver diseases and the pattern of vaccination. Therefore, to adjust the effect of these confounder variables, we entered all confounders in addition to BMI category in a linear regression model. Among all related variables with significant or borderline association with anti-HBs titer, only the presence of liver disease (*P*=0.003) was an independent predictor of determining the level of anti-HBs titer (standardized beta=0.264). With the substitution of quantitative BMI instead of categorical BMI in this model, only quantitative BMI remained as the independent predictor of the level of anti-HBs titer (standardized beta=-0.292, *P*=0.001).

## DISCUSSION

Control group (with normal BMI) used more immunosuppressive medications, had more percentage of liver diseases, were younger, had more percentage of autoimmune liver disease, and poorer vaccination history. Among these variables, only the presence of liver diseases and the higher numbers of vaccination times were correlated with the higher titer of anti-HBs. BMI had also a reverse moderate correlation with the level of anti-HBs titer. Therefore, the liver disease and the pattern of vaccination were considered as confounders in evaluating the association between BMI and anti-HBs titer. By adjustment of these variables, the presence of liver disease and lower BMI remained as independent predictors of anti-HBs titer in two separate models. This observation shows that obese individuals have significantly lower response to hepatitis B vaccination. We should, therefore, consider some strategies to overcome such weak response by increasing the subgroup of population.

Despite the unsuitable situation of controls in view of immunosuppression medication usage, liver disease, and vaccination history, they had a higher level of anti-HBs titer, showing that BMI is a significant major risk factor for poor response to hepatitis B vaccination. Its effect on anti-HBs titer can even be higher from the impact that acquired in our study because of unsuitable situation of our controls in response to hepatitis B vaccination.

In Iran, most hepatitis B vaccines are from Cuba. A Cuban hepatitis B vaccine on 64 nurse’s aid students caused a protective level of anti-HBs in 42.2% (http://ebnesina.ajaums.ac.ir/browse.php?a_id=46&sid=1&slc_lang=en), and in 235 health care providers was 81.2%. It was also 92.3% in our control group when their mean age was 39 years and most of them were female. BMI≤25 kg/m^2^[9], older age (more than 39)[5,10,11], smoking[5,10], obesity[5,10-12], male gender[10,11], and increasing time interval after completing vaccine[5] were independent predictors of poor response to hepatitis B vaccination in similar previous studies. In our study, obesity was the only independent risk factor for such weak response. Other variables were not related to the hepatitis B vaccine response. Although higher BMI was associated with poor response to hepatitis B vaccination in univariate analysis, our multivariable analysis showed that BMI is not an independent risk factor for response to this vaccine. In other studies[5,6,12], multivariate analysis have shown that BMI<25 is an independent predictor of poor response to hepatitis B vaccine. In some previous studies[6,10,11], response to vaccination has usually been considered as yes/no (qualitative response). However, we have considered the quantitative level of anti-HBsAb, which showed a reverse association with BMI, a finding that has less often considered in literature. Moreover, we have studied associated factors and have a unique control group that was not comparable with relatively similar studies.

Similar to our previous study[13], we found here that the family history of liver disease was not associated with response to hepatitis B vaccine. However, one study has shown low response rate to hepatitis B vaccine in immunosuppressed patients with inflammatory bowel disease[14]. Other investigations have revealed that immunosuppressive regimens used by mothers do not affect the offsprings’ immune status[15]. In our study, immunosuppressive treatment was not a significant variable for response to hepatitis B vaccine. However, the subject is controversial and needs more sophisticated studies.

A very recent meta-analysis showed that a significantly decreased response to hepatitis B vaccine is associated with age ≥40 years, male adults, BMI≥25 kg/m^2^, being smoker, and adults with concomitant diseases[16]. Poor vascularity of fat tissue and subsequent slow mobilization and processing of antigen, longer reaching time to the circulation from fat tissue, denaturizing antigens in fat tissue, poorer drainage of adipose tissue in comparison with muscle due to lower blood supply and subsequent higher adverse effects in subcutaneous vaccination are all mentioned as mechanisms of weak response to hepatitis B vaccination and higher side effects in subcutaneous in comparison with intramuscular injection[17]. The same hypothesis can be considered in more obese cases in comparison with non-obese ones.

Longer needles have lower reactogenicity with the same immunogenicity when use for other vaccines such as combined diphtheria, tetanus, pertussis, and Haemophilus influenza, and a serogroup C meningococcal vaccine[18].

The injection of hepatitis B vaccine with usual syringe in morbid obesity cases may be equal to subcutaneous injection, which has been shown to produce lower seroconversion rate and even rapid decline of antibody response than intramuscular injection[19]. A published study in JAMA showed that women have greater deltoid skin-fold thickness than men and need longer syringe. Women with weight less than 60 kg, 60-90 kg, and more than 90 kg needed 16-, 25-, and 38-mm needle to ensure intramuscular administration, respectively[19]. Because of some degrees of immunodeficiency in obese cases, booster immunizations might be helpful in these cases[20]. General recommendation now available for needle length has shown that needle should be long enough to reach the muscle mass for intramuscular vaccines, which causes both less local complications and maybe higher immunization[21].

We have not checked the data of last vaccination, while it is known that anti-HBs are declined with time and may even become undetectable after several years. However, it remains for at least five and eight years in adults and children, respectively.[22] Cross-sectional nature of the study, and unawareness from BMI at the time of vaccination are among other shortcomings of the present study.

Many debates have been issued about the obesity and hepatitis B vaccine. Most investigations, as mentioned earlier, did not design their studies according to different BMI and have just evaluated BMI beside other probable risk factors and demographic and anthropometric variables.[5,6] However, our study has compared cases with different BMI in view of response to hepatitis B vaccination as its main goal. The control group in this study was selected from a referral center of hepatology clinic. Similar to our cases group (obese persons), the probability of both liver diseases and immunodeficiency is higher than general population. Therefore, such control group is superior to healthy, neighborhood, family members, or hospital controls because selected control group is more comparable with our cases.

The level of anti-HBs titer in obese cases is lower than the controls, even in the presence of important factors like male gender and older age as confounders. However, the presence of liver disease can be an important factor that significantly affects this relation. Hence, anti-HBs titer level in obese cases without liver disease is lower than non-obese cases without liver disease, and we therefore should use a suitable strategy to overcome this problem; For example, using longer needles for vaccination in these cases.
